# Axial Torsion and Gangrene: An Unusual Complication of Meckel's Diverticulum

**DOI:** 10.7759/cureus.6702

**Published:** 2020-01-19

**Authors:** Hassan Bin Ajmal, Zainab Majid, Faryal Tahir, Saima Sagheer

**Affiliations:** 1 General Surgery, Dow University of Health Sciences, Karachi, PAK; 2 Internal Medicine, Dow University of Health Sciences, Karachi, PAK; 3 Breast and General Surgery, Civil Hospital, Dow University of Health Sciences, Karachi, PAK

**Keywords:** meckel's diverticulum, torsion of meckel's diverticulum, gangrene, axial torsion, diverticular disease

## Abstract

Meckel’s diverticulum (MD), a congenital abnormality of the gastrointestinal tract, is usually found in the pediatric population younger than two years of age; hence, its incidence in adults is rare. Although MD is mostly clinically silent, in adults, it may present with intestinal obstruction and diverticulitis. The complications of MD include hemorrhage, perforation, enterolith formation, torsion, Littre’s hernia, ulceration and neoplasm. Among these, torsion is one of the rarely reported complications of MD. MD being attached to the ileal mesentery or umbilicus, presence of mesodiverticular band, and the length, breadth and base diameter of the diverticulum contribute as a risk factor for torsion. A similar clinical picture of acute appendicitis must be excluded.

We report a case of a 25-year-old male who presented with signs of intestinal obstruction in whom intraoperative finding of a torted MD with necrotic and twisted base was found upon emergency exploratory laparotomy.

## Introduction

Meckel’s diverticulum (MD) is a congenital abnormality of the gastrointestinal tract arising due to the persistence of the viteline duct, responsible for forming a connection between midgut and yolk sac in the fetal life [1]. The basis of this embryonic remnant was first described in 1806 by the German anatomist, Johan Friedrich Meckel [2]. MD follows the well-established “rule of 2,” according to which the incidence is found in 2% of the population usually younger than two years of age, with dimensions of about two inches and at a distance of two feet from the ileocecal valve [3]. Although MD is mostly clinically silent, it is a common term among pediatric surgeons as it may produce symptoms of rectal bleeding or intussusception in children, whereas adults, infrequently, may present with intestinal obstruction and diverticulitis among other rare symptoms [1,4]. The complications of MD include hemorrhage, perforation, enterolith formation, torsion, Littre’s hernia, ulceration and neoplasm [4,5]. Among these, torsion is one of the rarely reported complications of MD [6].

We report a case of a 25-year-old male who presented with signs of intestinal obstruction with intraoperative findings of a torted MD with necrotic and twisted base.

## Case presentation

A 25-year-old male with no known comorbidities, laborer by profession, was presented in emergency room with a chief complaint of acute, severe abdominal pain for seven days. Initially, the pain was localized in the left hypogastrium. Later, the entire hypogastrium was involved along with nausea, vomiting and mild undocumented fever. Following pain, abdominal distention and failure to pass stool and flatus were reported. Past medical and surgical history was unremarkable.

On physical examination, an anxious looking young male was lying on the bed with distress but conscious and oriented. He was tachycardiac, tachypnic and dehydrated. The vitals recorded were heart rate of 105 beats/min, blood pressure of 120/70 mmHg, respiratory rate of 28 breaths/min and temperature 99°F. A grossly distended abdomen with generalized tenderness but more towards the hypogastrium, mild rigidity and guarding was found along with tympanic percussion and absence of gut sounds. A digital rectal examination revealed collapsed rectal walls with the absence of feces. Examination of all other systems was unremarkable.

The patient was resuscitated with intravenous fluids. A nasogastric (NG) tube and urinary catheter were passed to measure the output. The NG tube collected around 800 mL bilious content while the patient maintained a good urine output.

Laboratory investigations revealed hemoglobin 13.8 g/dL [normal (N) = 13.8-17.2], total leukocyte count 12.5 x 109/L (N = 4-11), neutrophils 82% (N = 60-70) and platelets 315 x 109/L (N = 160-398). Total bilirubin was 1.24 mg/dL (N = 0.3-1). Renal function test and liver function test were found to be normal with no viral markers.

**Figure 1:**
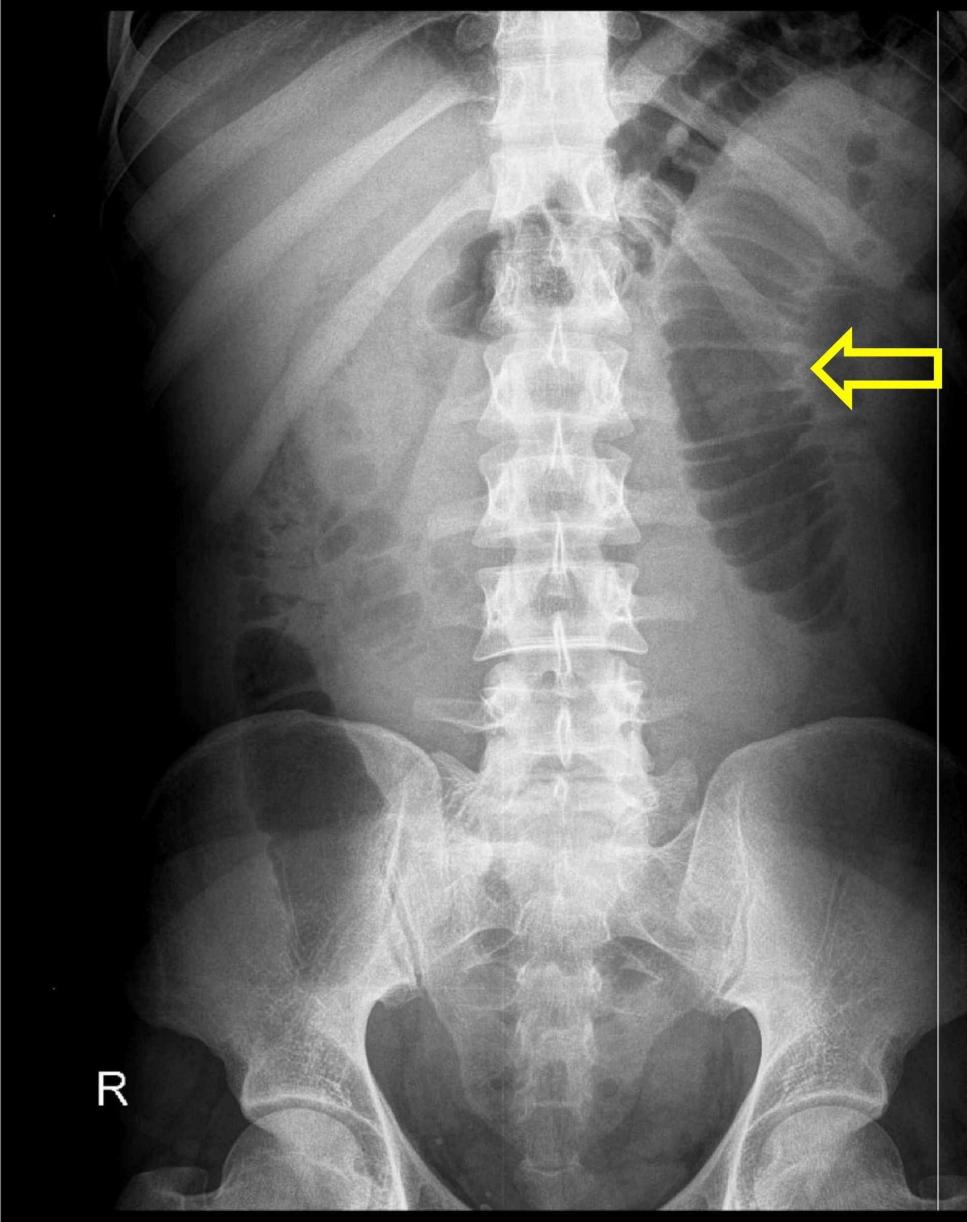
Abdominal X-ray showing dilated jejunum (yellow arrow) with no air in the rectum

An abdominal supine radiograph was ordered which showed dilated small bowel with no air in the rectum (Figure 1). No other radiological investigations were performed, and a clinical diagnosis of acute abdomen secondary to acute intestinal obstruction was made. The patient was prepared for emergency exploratory laparotomy.

**Figure 2:**
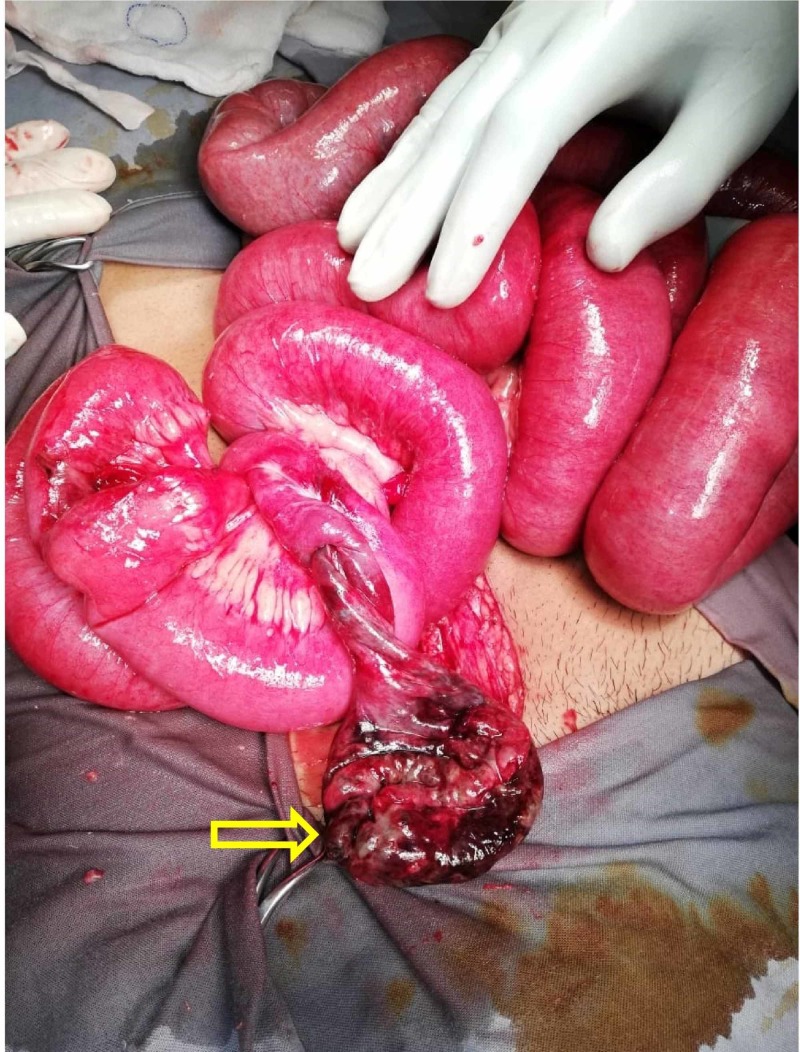
Dilated and necrotic MD with twisted bowel loop at its base (yellow arrow) MD: Meckel's diverticulum

Exploratory laparotomy, using a vertical midline incision, revealed 500 mL of serosanguinous fluid in the peritoneal cavity along with distended small bowel loops. Bowel run was done and on further exploration, 50 cm proximal to the ileo-cecal junction, a complete MD was found distended, necrotic and twisted around its base (Figure 2). Distended but viable bowel loops were found with no signs of visceral injury. The twisted MD was resected along with 5 cm of ileum proximal and distal to its base. An end-to-end seromuscular anastomosis using 2/0 vicryl suture was made to re-establish the continuity of small intestine. A thorough peritoneal washout was done followed by the insertion of pelvic drain.

Our patient went through uncomplicated recovery and was discharged on 12th postoperative day with advice of routine follow-up.

## Discussion

MD is a remnant of the viteline duct and is a type of true diverticulum, consisting of all the layers of the gastrointestinal wall (mucosa, muscularis and serosa). The viteline duct regresses during 6th to 10th week of fetal development but occasionally may persist as a blind loop located at the antimesenteric border of the distal ileum [2,4]. The wall of the MD is lined by intestinal mucosa similar to that of ileum but it is not rare for an ectopic tissue to be found. Most often, gastric and pancreatic tissues are present, 62% and 6%, respectively, with sparse incidence of duodenal and colonic tissues [1,7]. The presence of ectopic tissue influences the mode of management and the outcomes.

Patients with MD are widely asymptomatic but in 4%-7% of the cases, a possible complication leads to its detection [2]. A 2008 study showed merely 4.2% of patients presenting with symptomatic MD, while another study reported a figure of 9% supporting the idea of infrequent detection of MD [7]. Male gender is more prone to develop MD and usually occurs before the age of 10 years as the incidence decreases with advancing age; hence, it is rarely found in adults [5]. The presentation of MD varies depending on the underlying complication. The usual symptoms include abdominal pain with or without fever, distention, vomiting, constipation, bleeding per rectum and seldom chronic pain [8]. The most common complications in adult population are bowel obstruction in 40% of cases and peritonitis in 5%-19% cases which paints a similar picture to acute appendicitis, and hence a common differential for MD [8,9]. Hemorrhage predominantly occurs in pediatric age group in association with peptic ulceration, usually in the presence of ectopic gastric tissue found in 20%-55% of the cases [1,4]. Obstructive symptoms arise commonly due to inflammatory adhesions, neoplasm, incarcerated hernia, strictures, enterolith, intussusception, torsion and volvulus [10].

Torsion is one of the rarest complications of MD, and only a few reports exist that discuss this complication in adults [4]. Although the exact mechanism of developing torsion is unclear, some explanations have been put forward. MD is sometimes attached to the ileal mesentery or umbilicus, and the presence of mesodiverticular bands makes it susceptible to torsion [11]. Another contributing factor associated with torsion is the length, breadth and base diameter of the diverticulum, with elongated type of MD having a narrow base being more at risk for torsion [12]. The MD in our case measured 12 cm which underwent axial torsion around its narrow base compromising the blood supply and eventually becoming necrotic. In cases of torted MD, pain is usually localized to right lower quadrant but the site may vary. Our patient complained of pain in the left hypogastrium with symptoms supporting intestinal obstruction and no signs of appendicitis. Since the abdominal x-ray showed no air in the rectum with dilated jejunum, it was decided to perform an exploratory laparotomy to further examine and treat the cause.

Preoperative diagnosis of MD is a serious challenge. Nowadays, several imaging modalities are used which include x-ray, ultrasound, computed tomography (CT) and magnetic resonance imaging, but have proved to be of little diagnostic value [7]. Plain x-ray is good for revealing intestinal obstruction and pneumoperitoneum along with the presence of enteroliths or gas-filled diverticulum [1]. CT scan may also show gas filled viscera but its use is limited due to difficulty in distinguishing MD from normal bowel. Angiography of arteria mesenterica superior is crucial for detecting the site and reason of hemorrhage in complicated cases of MD [5]. Technetium-99m scan also provides great diagnostic clue for MD as the radionucleotide is taken up by the heterotropic gastric mucosa [10]. The mainstay of treating symptomatic MD remains surgical resection. However, operating an incidentally found MD is still debated.

## Conclusions

MD is a rare cause of acute abdomen due to its low incidence in general population. MD infrequently undergoes axial torsion rendering the bowel loops susceptible to ischemia and necrosis. The risk of torsion greatly increases with the increased length and narrow base of the MD along with presence of mesodiverticular bands. Often, this condition is misdiagnosed as appendicitis and provides a diagnostic challenge. Imaging studies like x-ray are of little diagnostic value, and exploratory procedures provide a better picture. Prompt diagnosis and management via surgical resection in most cases leads to uncomplicated recovery; however, intervening an asymptomatic MD is still debated.
